# The role of the General Practitioner in weight management in primary care – a cross sectional study in General Practice

**DOI:** 10.1186/1471-2296-9-66

**Published:** 2008-12-15

**Authors:** Marlene Tham, Doris Young

**Affiliations:** 1 University of Melbourne, Department of General Practice, 200 Berkeley Street, Carlton, Victoria 3053, Australia

## Abstract

**Background:**

Obesity has become a global pandemic, considered the sixth leading cause of mortality by the WHO. As gatekeepers to the health system, General Practitioners are placed in an ideal position to manage obesity. Yet, very few consultations address weight management. This study aims to explore reasons why patients attending General Practice appointments are not engaging with their General Practitioner (GP) for weight management and their perception of the role of the GP in managing their weight.

**Methods:**

In February 2006, 367 participants aged between 17 and 64 were recruited from three General Practices in Melbourne to complete a waiting room self – administered questionnaire. Questions included basic demographics, the role of the GP in weight management, the likelihood of bringing up weight management with their GP and reasons why they would not, and their nominated ideal person to consult for weight management. Physical measurements to determine weight status were then completed. The statistical methods included means and standard deviations to summarise continuous variables such as weight and height. Sub groups of weight and questionnaire answers were analysed using the χ^2 ^test of significant differences taking p as < 0.05.

**Results:**

The population sample had similar obesity co-morbidity rates to the National Heart Foundation data. 74% of patients were not likely to bring up weight management when they visit their GP. Negative reasons were time limitation on both the patient's and doctor's part and the doctor lacking experience. The GP was the least likely person to tell a patient to lose weight after partner, family and friends. Of the 14% that had been told by their GP to lose weight, 90% had cardiovascular obesity related co-morbidities. GPs (15%) were 4th in the list of ideal persons to manage weight after personal trainer

**Conclusion:**

Patients do not have confidence in their GPs for weight management, preferring other health professionals who may lack evidence based training. Concurrently, GPs target only those with obesity related co-morbidities. Further studies evaluating GPs' opinions about weight management, effective strategies that can be implemented in primary care and the co-ordination of the team approach need to be done.

## Background

Obesity is considered the sixth leading cause of mortality in the world today according to the World Health Organization, highlighting its transformation into a global health pandemic [1]. It is second only to smoking in the number of preventable deaths in Australia it causes, and the levels of obesity and its co-morbidities are rising every year [2,3].

As the gatekeepers to the health care system, General Practitioners (GPs) can play a vital role in addressing obesity in the consultation. 80% of the Australian population consult their GP throughout the course of the year [4]. Yet with more than 60% of the population [3,5] considered overweight or obese, the question remains why presentations to GPs for weight management make up less than 1% of total consultations [4].

There is limited literature that explores this question. A recent Australian study suggested that 78% of patients felt that GPs had a role in weight management but less than half thought that GPs would be able to spend enough time to provide effective weight loss advice [5]. A number of studies from the USA indicate that patients were disappointed with primary care weight management and wanted substantially greater effective involvement by their family physician [6-11]. However, there are no studies on when and how often GPs bring up weight during the consultation, the impact of having an obesity related co-morbidity on weight management for both doctor and patient and who is the patients' perceived "ideal person" to seek help and advice for losing weight.

This cross-sectional study's objective is to explore further reasons why patients attending GP appointments are not engaging their GP for weight management, and their perception of the role of the GP in managing their weight. It is hoped that the findings of this exploratory study will help improve and direct primary care weight management from both the patients' expectations and in GP consultations themselves.

## Methods

367 participants aged between 18 and 64 were recruited from the waiting rooms of three Melbourne (Australia) general practices over a three month period from February to April 2006 for this cross-sectional study. The three practices varied in size, socio-economic status and were a mix of private and completely government funded billing methods. All three practices had similar access to weight management options offered by the community. Ethics was obtained from the University of Melbourne ethics committee. In three nominated sessions each week, the reception staff offered all eligible participants an information sheet and consent form to participate in the study. Those who consented proceeded to fill out a self administered tick box questionnaire in the waiting room which included:

1) Basic demographics (age, gender, co-morbidities).

2) If they had a weight problem in the last 2 years, who had asked them to lose weight.

3) If they believed their GP had a role in weight management, how likely would they bring up weight management with their GP and reasons why they would not.

4) Their nominated ideal person to consult for weight management.

This was followed by physical measurements of weight, height and waist circumference measured by the principal investigator (MT) to determine weight status by calculating body mass index (BMI, kg/m2) [underweight (< 18.5), healthy weight (18.5 – < 25), overweight (25.1 – < 30), and obese (> 30.1)]. Weight was measured using scales correct up to 0.1 kg, height was measured with a wall mounted height measurer and waist circumference was taken at the part of the trunk located midway between the lower costal margin and the iliac crest while the person was standing as per the WHO Guidelines [12]. Obesity related co-morbidities were defined as those related to the cardiovascular system. They were hypertension, hypercholesterolaemia, type 2 diabetes, myocardial infarction, angina and stroke [12]. Patients who had intellectual or physical disability or language barriers that would significantly impair ability to participate in the study, and those who were acutely unwell were excluded from the study.

The statistical methods included means and standard deviations to summarise continuous variables such as weight and height. The BMI was cross tabulated with questions about their attitudes towards weight and the role of the GP. The χ^2 ^test was used to analyse sub groups for significant differences taking p < 0.05. Categorical variables was summarised using frequencies and percentages. The analysis was done using SPSS version 14.

## Results

### Sample Size

A total of 425 patients were approached with 394 meeting the inclusion criteria. 367 participants consented to participate (91%) consisting of 143 females and 124 males. The age distribution showed no signs of non-normality. There was a similar rate of obesity related cardiovascular co-morbidities when compared to the Australian general population with 28% diagnosed with hypercholesterolaemia, 27% with hypertension, 8% with diabetes and 3% with angina. The percentages of patients who had experienced a cardiac event were heart attack (5%) and stroke (4%). The total percentage of patients who had at least one co-morbidity was 38%, those that had two were 19% and those with three was 8%.

### Physical Measures

42% of the sample size was overweight, 28% were obese and 8% were very obese, meaning the total of patients who had a BMI greater than 25 was 78%. Males (73%) were significantly more overweight and obese than females (64%) (p < 0.05). There was no significant difference between the general Australian population levels of overweight and obese when compared to the National Heart Foundation data, 2005 [12]. For males, as the age increased so did the levels of obesity with 14.3% of males in the 55–64 year old age group considered very obese. The percentage of overweight females also increased with age.

Refer to Table 1 below for full age and gender BMI figures.

**Table 1:** % of Weight Status of Gender and Age

**Gender**	**BMI category**	**Age**					**Total**
		18–24	25–34	35–44	45–54	55–64	

**Female**	underweight	8	4	3	3		3

	healthy weight	48	32	37	27	21	22

	overweight	18	35	21	45	45	33

	obese	26	29	39	25	34	33

							100

**Male**	underweight						

	healthy weight	38	28	18	20	18	26

	overweight	35	47	59	50	37	45

	obese	27	24	22	30	45	29

							100

### Overweight and Obese status as related to co-morbidities

The percentage of patients that had at least one cardiovascular obesity related co-morbidity also increased with age. Of the 78% who were overweight or obese, 64% had at least one co-morbidity. 18–24 year olds who were considered to have a healthy weight had no cardiovascular obesity related co-morbidities compared to the oldest age band 55–64 year olds who were considered obese and who had the highest percentage of at least one co-morbidity. These percentages were consistent with the BEACH (Bettering the Evaluation and Care of Health) Study date of Australian General Practice consultation [4]. Hence both age and weight status influence the likelihood of a having a cardiovascular obesity related co-morbidity.

See table 2 below for full results.

**Table 2:** The percentage of patients defined by weight status in each age group that had at least 1 cardiovascular obesity related co-morbidity

**Weight/Age**	**18–24**	**25–34**	**35–44**	**45–54**	**55–64**
**Healthy**	0%	5%	11%	16%	22%

**Overweight**	9%	18%	35%	49%	64%

**Obese**	7%	30%	46%	68%	78%

### Who Tells Patients to Lose Weight?

In the last 2 years, only 29% of the sample were told they needed to lose weight despite 66% being overweight or obese. 55% of those who were overweight were not told they needed to lose weight by anyone compared to 42% of those who were obese. Only 45% of patients with a co-morbidity were told to lose weight. Of those who had been told to lose weight in the last 2 years, it was the partner (45%) who was the most likely person to tell the patient to lose weight, followed by family member (25%), then friend (16%) with doctor last (14%).

### The GP's role in weight management

**Figure 1:**
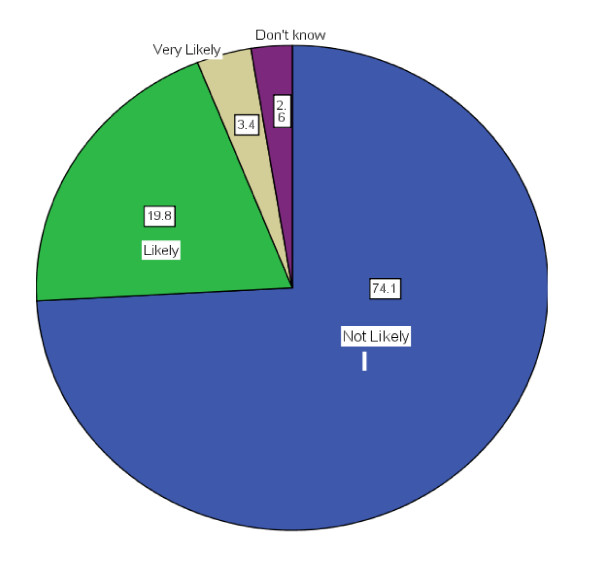
The likelihood of patients bringing up weight management during the GP consultation.

70% of participants did believe that their GP had a role in managing their weight. However, 74% of patients stated that they were not likely to bring up weight management when they visit the doctor compared with 19.8% who were likely (see figure 1). When comparing those with at least one obesity related co-morbidity with those who had none, patients were three times more likely to bring it up. (Pearson's χ^2 ^(3) = 2.468 Pr = 0.002). There was no gender difference in those who were likely to bring up weight management with their GP (Pearson's χ^2 ^(3) = 4.7644 Pr = 0.19). For those who did not see their GP as having a role, the most common reasons given were: "It is none of their business", "They are too busy with other medical issues to be burdened with weight" and "Not enough time in consult"

Of the 14% that had been told by their GP to lose weight, 90% had obesity related co-morbidities and the 10% with no co-morbidities who were told were in the obese category. No patients in the overweight group were asked to lose weight.

**Figure 2:**
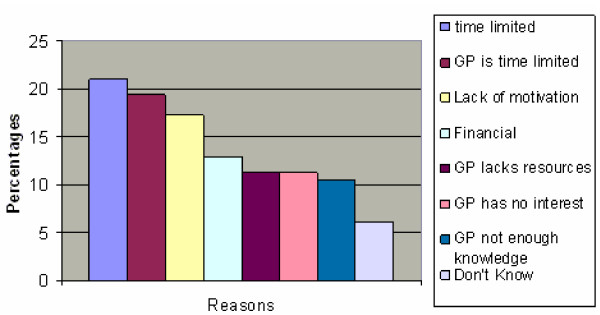
Reasons why weight was not brought up during the consultation.

For those patients who believed they were overweight or obese in the past or believe they are currently overweight or obese, factors which have stopped them bringing up weight management with their GP are listed below in figure 2. Time limitation on both the patient's and doctor's part was the most commonly reported barrier, followed by lack of motivation.

### Ideal person nominated to help lose weight

**Figure 3:**
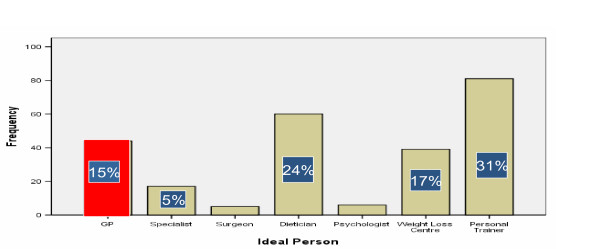
Ideal person nominated to help lose weight.

GPs were considered 4^th^, in the list of most ideal persons for weight management. Personal trainer was considered the most ideal person followed by dietitian. The surgeon was the least chosen ideal person with less than 1% of responses. For those with at least one co-morbidity, GPs were chosen 30% of the time. See figure 3.

## Discussion

This study is novel in its attempts to determine why there are so few consultations for weight management when more than 60% of the population is overweight or obese. The BEACH study of Australian general practice activity indicates that less than 1% of GP consultations are primarily for weight management when the country is facing an obesity crisis [4]. This study also attempts to evaluate if there is a difference between patients' perception of the GP's role and the GP's activeness in bringing up weight during the consultation if the patient has already has an obesity related disease.

The results of this exploratory study may indicate a general acceptance of larger body size and attitudes from both patient and doctor and that addressing weight and losing weight may still be a topic that is not brought up in medical consultations despite the obvious negative health effects. This may also be a result of lack of communication between patient and doctor or a lack of weight management skills and confidence by the doctor.

One of the issues that may be highlighted is that of those in the sample that the GP did tell to lose weight, only 10% did not have any established cardiovascular obesity related co-morbidities but were obese. Yet, it is well established that reducing weight is an effective primary prevention strategy of many diseases [1-3,12,13] and that the direct cost of obesity to the economy has been said to be $2.4 billion a year and the indirect costs as high as related $9 million [14]. It can be postulated that GPs can contribute to reducing this burden by preventing the development of these diseases as opposed to acting when the disease is already present. Yet, this study indicates that GPs are reluctant to bring up weight during consultations unless they had an associated illness. This was supported by similar studies which found that less than half of obese adults reported being advised to lose weight by their GPs and that communication about weight was only targeted to patients with higher BMIs and obesity-related co-morbidities [1,6-9].

Another possible reason for the low number of consultations with GPs for weight management is that patients do not have confidence in their GPs, despite more than two thirds stating that believed that GPs have a role. A number of studies from the USA [6-11] and overseas [12,13] indicate that patients were disappointed with primary care weight management and wanted substantially greater effective involvement by their GPs [7-10]. Similarly, this study shows that less than 20% of patients would actually bring up their weight in the consultation. The fact that many cardiovascular obesity related co-morbidities such as hypertension, hypercholesterolaemia, diabetes, heart disease and strokes encompass a wide range of medical specialities and allied health providers, emphasises the need for a team approach. As this study shows that patients prefer personal trainers and dieticians above medical practitioners (GPs, surgeons, physicians) as managers of weight, there is obvious benefit in establishing a multi – disciplinary approach for weight management which may involve partners and family, allied health and medical practitioners. There appears to be no established facility in Australia that embraces this approach for overweight and obese patients who do not have an existing disease.

It appears that there is a reluctance in both parties to discuss weight management in GP consultations. There is certainly a mismatch between the urgency to address the obesity epidemic and the creation of interventions based in primary care. With international studies [8-12] also indicating a lack of confidence in GPs' ability to manage weight, perhaps education should be targeted at GPs to improve their weight management skills and knowledge. A first step would be to evaluate GPs' perception of their overweight patients and also to research their confidence and ability to manage obesity.

Consequently, there has been much discussion regarding an urgent need to improve the standards of obesity management in primary care [15-17]. A survey done by 756 Australian GPs in 2000 indicated that they thought the prevention and management of obesity needs addressing [16]. Effective communication skills, particularly for sensitive issues such as weight, may need to be improved in GPs so that they may improve their confidence in discussing weight issues with patients. Many GPs also cite lack of time, training, resources, staff support, adequate reimbursement and fear of negative patient reactions as common reasons why they fail to treat obesity effectively [7,8,16-18]. The establishment of a team approach to obesity may be one way to address this problem. The Counterweight Project, a UK based primary care weight management has shown some promising results and may used as a model [19].

This study's limitations include not investigating factors such as motivation, and the impact of other illnesses as a contributing factor to the low consultation rate. Also, this was a small sample involving only three general practices and the findings may not be generalised to a wider population. The study needs to be extended to other GP practices including those in the rural community for confirmation.

## Conclusion

It is becoming increasingly apparent that obesity is a complex problem which needs to be addressed urgently. GPs can play a vital role in managing obesity as the gatekeepers to the health system. There are very few recent Australian studies looking at the role of the GP [2,16]. There are moves being made by the Australian Health System for a team approach in primary care for the management of obesity. This is with the introduction of the Enhanced Primary Care Program (EPC) which can fund the GP and the team in treating complex and chronic conditions such as those associated with obesity related co-morbidities. But a way to co-ordinate this in primary care and in "multi-disciplinary share clinics has not been established. Effective communication skills and training in managing weight are other key issues that must be addressed if primary care weight management is to be improved. Hence, further studies evaluating the GPs opinion on weight management, effective strategies that can be implemented in primary care, the co-ordination of multi-disciplinary clinics for weight management and the impact of the team approach with the involvement of the GP need to be done.

## Competing interests

The authors declare that they have no competing interests.

## Authors' contributions

Both MT and DY conceived the study and participated in its design and coordination and helped to draft the manuscript. MT was the researcher who participated in the gathering of data at the practices, and performed the statistical analysis using SPSS. DY was the supervisor of the project. Both authors read and approved the final manuscript.

## Pre-publication history

The pre-publication history for this paper can be accessed here:


